# The role of MiRNA‐433 in malignant tumors of digestive tract as tumor suppressor

**DOI:** 10.1002/cnr2.1694

**Published:** 2022-08-17

**Authors:** Jie Tang, Jiawei Chen, Yongqiang Wang, Shaobo Zhou

**Affiliations:** ^1^ General Surgery The Second Affiliated Hospital of Bengbu Medical College Bengbu China

**Keywords:** gallbladder carcinoma, malignant tumors of digestive tract, miRNA‐433, tumor suppressor

## Abstract

**Background:**

MicroRNAs (miRNAs) are a class of short non‐coding RNAs with a length of approximate 22 nuclei acids that can be expressed both as an oncogene and tumor suppressor gene in human cancers. MiRNAs can participate in the post‐ transcriptional regulation of gene expression, and regulate the several cancer‐related processes, including proliferation, apoptosis, metastasis, etc.

**Recent findings:**

Expression of miRNA‐433 has been reported to vary in different tumors and affected by various factors. We have summarized the different previous studies and found that miRNA‐433 can significantly inhibit the growth of the cancer cells not only in malignant tumors of the digestive tract, but also in lung cancer, breast cancer, cervical cancer, ovarian cancer, bladder cancer, renal carcinoma, glioma, retinoblastoma and osteosarcoma.

**Conclusion:**

When the expression of miRNA‐433 was up‐regulated, the proliferation, metastasis and invasion abilities of the malignant tumor cells were significantly inhibited. At the same time, the potential mechanisms through which miRNA‐433 can suppress the growth and metastasis of the cancer cells were found to be basically the same, and involved modulation of the specific signaling pathways or target genes in the malignant tumors. Overall, it can be concluded that miRNA‐433 can serve as potential and valuable therapeutic target.

## 1 INTRODUCTION

**FIGURE 1:**
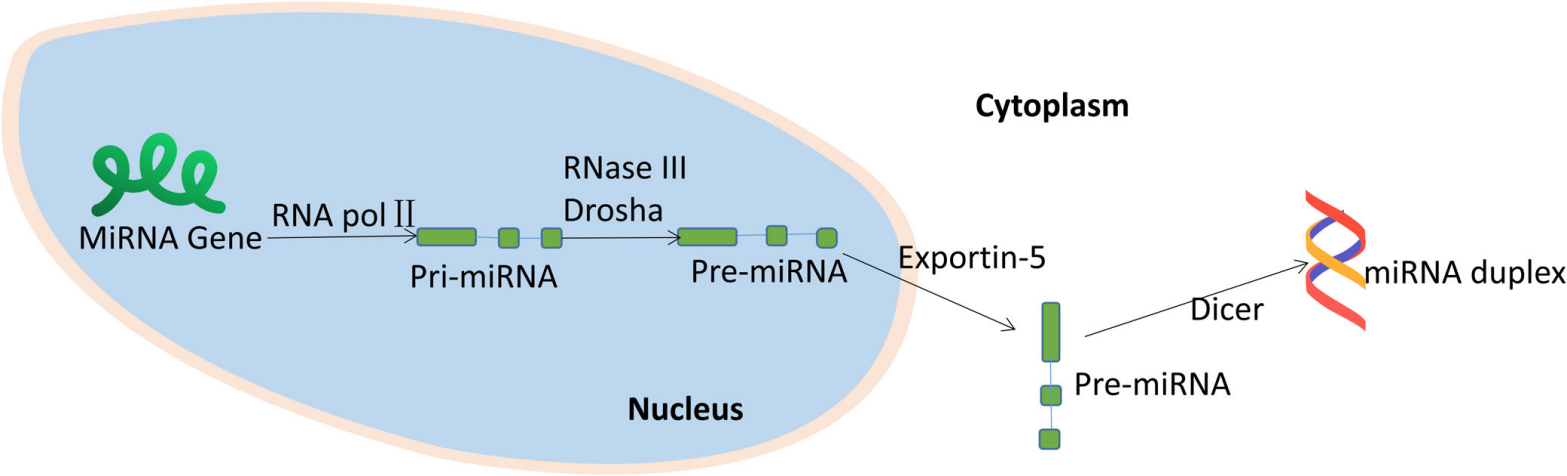
miRNA biogenesis in animals:MiRNA genes are typically transcribed by RNA polymerase II into primary RNA (pri‐miRNA), which is cleaved into the precursor miRNA (pre‐miRNA) by nuclear RNase III Drosha, and then exported into the cytoplasm by exportin‐5. In the cytoplasm, pre‐miRNA is cleaved by Dicer into the miRNA duplex

MicroRNAs (miRNAs) are short non‐coding RNAs with a length of about 22 nuclei acids, which can effectively regulate the gene expression in the transcription of plants and animals. The first miRNA was discovered in 1993,^1^ and according to the miRDB database, a total of 2656 microRNAs with targets have been identified in humans until 2019. It is well known that the central dogma of molecular biology can be simply summarized as: DNA ➔ RNA ➔ protein.^2^ In animals miRNAs genes are usually transcribed into the primary RNA, followed by cleavage into the precursor miRNAs and finally exported to the cytoplasm (Figure 1). MiRNAs have been reported play a pivotal role in the various biological processes, and their abnormal expression has been related to several diseases, including cancers.^3^, ^4^ miRNAs regulate mRNA transcription and thereby regulate the downstream protein expression.^5^

A tumor that can develop in the mouth, esophagus, stomach, intestine, liver, pancreas and gallbladder is defined as a tumor of the digestive tract. Among the various malignant tumors worldwide, the number of cases of gastrointestinal malignancies is extremely high. Colorectal cancer, gastric carcinoma, esophageal cancer and liver cancer rank in the top 10 in the tumor incidence rate. The 2020 Global Cancer Statistics show that these four cancers accounted for 23.4% of cancer incidence and caused 30.9% of all cancer‐related deaths worldwide. It has been found that incidence rates have increased, but mortality rates have decreased compared to 2018.^6^, ^7^, ^8^, ^9^ The World Cancer Research Fund/American National Cancer Institute research indicated that gastroenteric cancer was closely related to different modifiable risk factors, including drinking, smoking, diet and obesity.^10^, ^11^, ^12^, ^13^, ^14^ Since gastroenteric cancer is generally occult and does not display specific symptoms in the early stage, it is usually diagnosed in the middle and late stages,^15^, ^16^ thereby cauisng the treatment passive and limited. Therefore, the prognosis is rather poor.

**FIGURE 2:**
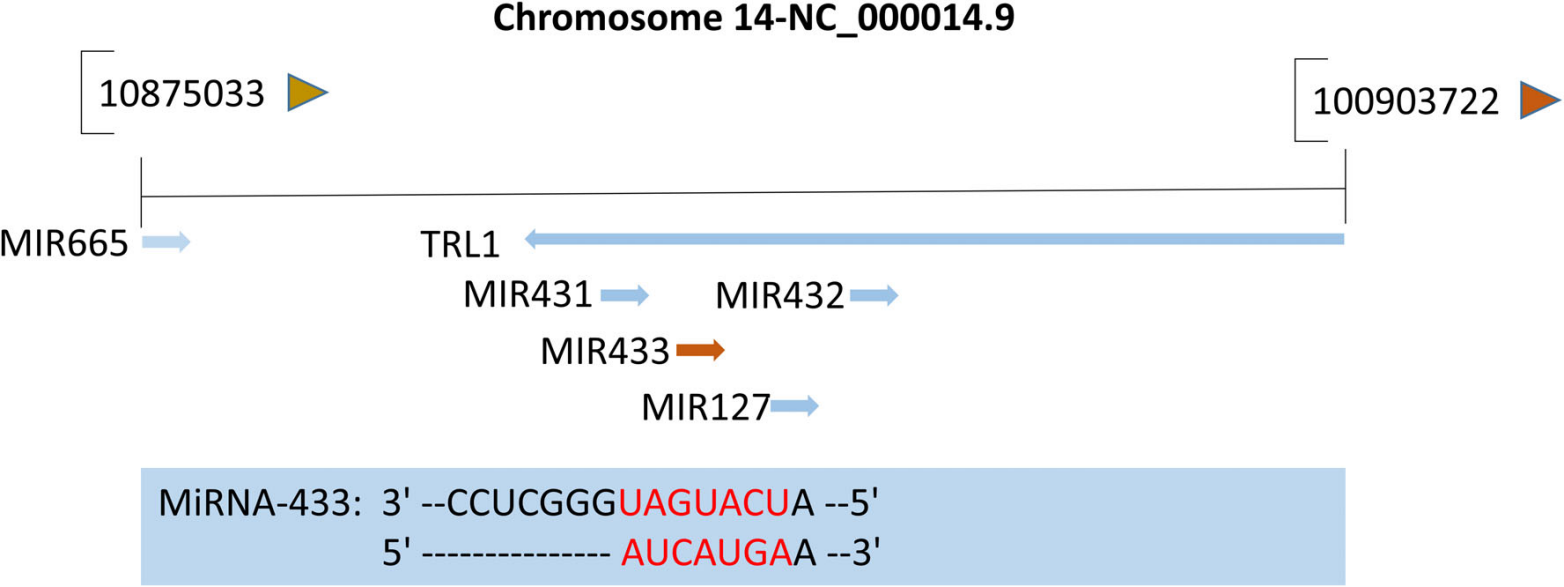
MiRNA‐433 gene location(Information from National Center for Biotechnology Information(NCBI)database). The red standard indicates the seed sequence of miRNA‐433 that binds to the various target genes in related malignant tumors of the digestive tract

**FIGURE 3:**
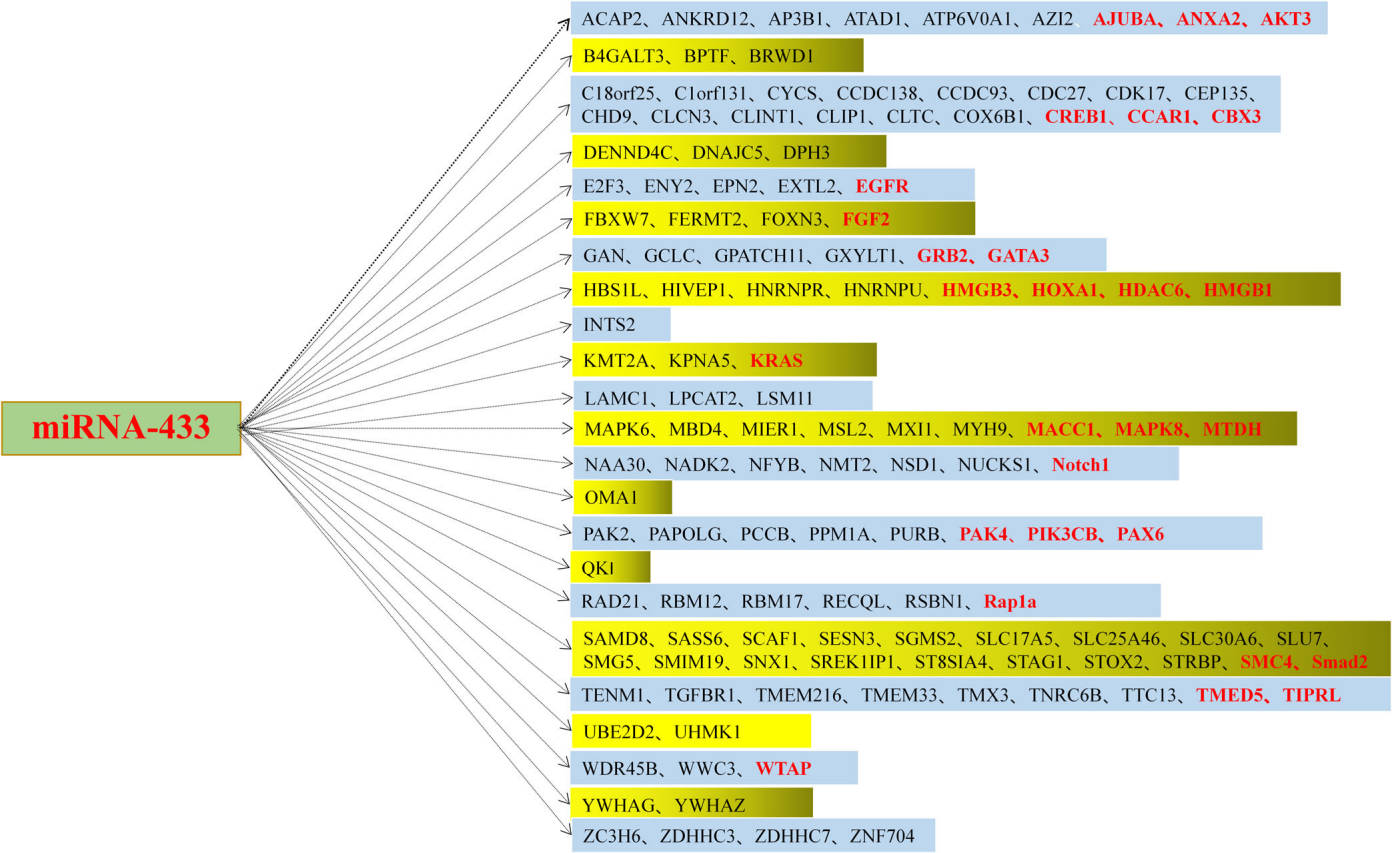
The predicted target genes of miRNA‐433, The red font indicates that the important role of the target gene in related cancers has been confirmed

With the in‐depth study of miRNAs, their roles in diseases have gradually become clear. For example,miRNA‐9‐5p can attenuate the migration ability of the human gastric cancer cells by inhibiting the expression of tumor necrosis factor alpha‐induced protein 8‐like 3(TNFAIP8L3).^17^ MiRNA‐552 can effectively promote laryngeal cancer progression by binding to *P53*.^18^ MiRNA‐217 can inhibit colorectal cancer progression through the *MAPK* signaling pathway.^19^ As a member of miRNAs，MiRNA‐433 plays an essential role in the progression of various malignant tumors. It is located in the human chromosomal region 14q32.2 (Figure 2)and participates in the regulation of tumors by binding to the various target genes. We have depicted the relevant targets through TargetScan and Mirranda (Figure 3). According to the NCBI database, miRNA‐433 is associated with 19 cancers. Hence, detailed studies deciphering the potential relationship between miRNA‐433 and diseases can significantly improve the understanding of mechanisms underlying gene expression regulation in the higher eukaryotes, thereby facilitating miRNA‐433 to serve as a novel biological marker for disease diagnosis.

## 2 MIRNA‐433 EXPRESSION LEVELS IN TUMORS: DOWNREGULATION

Because of enormous progress in research related to miRNA biology, some specific miRNAs have been discovered, and these miRNAs play an important role in both the occurrence and development of diseases. MiRNA‐433 is among one of them and we found that miRNA‐433 was downregulated in many malignant tumors, implicating its significant tumor suppressive role—the focus of this review. For example, Ting Li et al. by using Quantitative Real‐Time Polymerase Chain Reaction (RT‐qPCR)demonstrated that the expression of miRNA‐433 was significantly reduced in esophageal cancer as compared with the normal and adjacent tissues.^20^ This finding was not only validated in esophageal cancer, but also in gastric cancer,^21^ colorectal cancer,^22^ liver cancer,^23^ bile duct cancer,^24^ and pancreatic cancer.^25^

Moreover, in 2021, Yu Shui Ma et al. found that the level of miRNA‐433 was significantly down‐ regulated in the hepatocellular tissues as well as the cell lines (Hep3B， MHCC97H)and overexpressed miRNA‐433 exhibited potential tumor suppressive effects.^26^ In 2013, Ouy Ang Y Ang et al. reported that compared with the normal gastric mucosa, the expression level of miR‐433 was significantly down‐regulated and played a regulatory role in the occurrence and development of gastric cancer. The changes in expression of miRNa‐433 provided the basis for the diagnosis of the gastric cancer.^27^

In 2018, Li Yan et al. showed that miR‐433 level was down‐regulated in both the colorectal cancer tissues and cell lines (SW480, SW620, LoVo, HCT116, LS174‐T, Caco‐2, DLD‐1, RKO, and SW1116). Thus, miR‐433 can exhibit obvious tumor suppressive effects both under in vitro and in vivo settings.^28^ Of course, the malignant tumors in which miRNA‐433 expression has been found to be altered are far more than above discussed ones, and all have been summarized in Table 1. These findings clearly indicated that the down‐regulation of miRNA‐433 expression could serve as an important factor in the occurrence and development of the malignant tumors, which deserves further investigation.

**TABLE 1:** The various studies related to miRNA‐433 and malignant tumors of the digestive tract in the last 10 years

Tumor type	tumor suppressor	Downstream factors	Upstream inhibitory factors	Role of miRNA‐433
Oral cancer	+	*GRB2/MAPK*	/	A potential target for HDPC repair and regeneration^29^
	+	*HDAC6*	/	Inhibition of tumors through targeting *HDAC6* ^30^
	+	*PAK4*	*Linc01234*	*Linc01234* promotes OSCC progression through the miRNA‐433/*PAK4* axis^31^
Esophageal cancer	+	*GRB2*	/	Inhibition of the progression of ESCC through targeting *GRB2* ^32^
	+	*REV3L*	*Circ_0023984*	*Circ_0023984* promoted ESCC progression through the miRNA‐433/*REV3L* axis^20^
	+	*HMGB1*	*CircLPAR3*	*CircLPAR3* promoted ESCC progression by inhibiting miRNA‐433/*HMGB1* axis^33^
gastric carcinoma	/	/	/	Providing a basis for the diagnosis of GC^27^
	+	*KRAS*	/	Inhibition of cancers through *KRAS* ^21^
Colorectal cancer	+	*ANXA2*	*LINC00460*	*LINC00460 i*ncreased *ANXA2* expression by inhibiting miRNA‐433^34^
	+	*HOXA1*	/	Inhibition of the progression of CC through targeting *HOXA1* ^22^
	/	*MACC1*	/	Inhibition of the progression of CC through targeting *MACC1* ^35^
	+	*CyclinD1/CDK4*	/	Inducing G1‐S cell cycle arrest, suppressing *cyclinD1* and *CDK4* expression^36^
	+	*CREB1*	/	Inhibition of CC through targeting *CREB1* ^28^
Liver cancer	+	*CREB1*	/	Involvement in the metastasis of HCC through *CREB1* ^37^
	+	*AKT*	*KDM5A*	*KDM5A* increased *AKT* expression by inhibiting miRNA‐433^26^
	+	*CBX3*	*LINC01006*	*LINC01006* promoted HCC progression through the miRNA‐433/*CBX3* axis^23^
Cholangiocarcinoma	+	*HDAC6/MAPK*	/	Identification of the novel target of CCA^24^
Pancreatic cancer	+	*PAK4*	*LINC00657*	*LINC00657* promoted PDAC progression by targeting miRNA‐433/*PAK4* axis^25^
Nasopharyngeal carcinoma	+	*SCD1*	*HIF‐ 1α*	*HIF‐1α* regulated the progression of NPC through miRNA‐433/*SCD1* axis^38^

Abbreviations: *AKT*, Serine/threonine kinase; *ANXA2*, Recombinant Human Annexin A2; CBX3:Chromobox protein homolog 3; *CDK4*, Cyclin‐dependent kinase 4; *GRB2*, growth factor receptor‐bind protein 2; *HDAC6*, histone deacetylase 6; *HOXA1*, homeobox A1; *HMGB1*, high‐mobility group box 1; *KDM5A*, lysine‐specific demethylase 5A; *KRAS*, Kirsten rat sarcoma viral oncogene; *MAPK*, mitogen‐activated protein kinase; *PAK4*, activated kinase 4; *REV3L*, protein reversionless 3‐like; *MACC1*, metastasis associated in colon cancer 1.

## 3 ANTI‐TUMOR EFFECTS OF MIRNA‐433

### 3.1 Inhibition of proliferation, invasion, and migration

It has been established from above discussed reports that miRNA‐433 can primarily exhibit tumor suppressive effects. In order to understand its mechanisms of action in cancer biology, researchers have analyzed the role of miRNA‐433 in detail by upregulating miRNA‐433 expression in different cancers, and finally found that miRNA‐433 exhibited significant cancer suppressive effects.

A number of the previous studies have shown that the anti‐neoplastic action of miRNA‐433 is mainly manifested in three aspects: Inhibition of proliferation, invasion and migration of the cancer cells. For instance, In 2014, Xiaochun Wang et al. reported that restoring miRNA‐433 expression in oral squamous carcinoma cells can significantly inhibit the proliferation, invasion and migration of the tumor cells.^30^ Similarly, in 2020 Wen Jiang et al. found that in esophageal cancer, the various hallmarks such as cell proliferation, migration and invasion were effectively hindered by miRNA‐433.^33^ Even, miRNA‐433 has been linked to chemotherapy response of the gastric cancer and might be used as a potential serum marker to predict the efficacy of cisplatin in gastric cancer patients.^39^

Obviously, miRNA‐433 has been recognized to inhibit the proliferation, invasion and migration of the tumor cells, and these studies might provide new strategies for cancer treatment. MiRNA‐433 can also serve as potential predictive or prognostic biomarker in cancer management.

### 3.2 Proapoptotic role

The tumor suppressive effects of miRNA‐433 are not only reflected in the inhibition of tumor cell proliferation, migration and invasion, but also its ability to induce cancer cell apoptosis. For instance, in 2015, Jiaxin li et al. up‐regulated the expression of miRNA‐433 in the colorectal cancer cells and found that the up‐regulated miRNA‐433 could effectively reduce the survival rate of the cancer cells and promote apoptosis by down‐regulating *MACC1*.^35^ In 2019, Shasha Bi et al. reported that in the pancreatic cancer, miRNA‐433 promoted apoptosis while inhibiting the proliferation, migration, and invasion of the tumor cells.^25^ Moreover, similar apoptosis‐promoting effect was also found in other cancers, and these findings led to further confirmation of tumor suppressive actions of miRNA‐433.

### 3.3 Inhibition of colonization, EMT, and induction of ciliogenesis

In 2018, Mansini Adrian P et al. reported that targeted down‐regulation of Exportin‐5 expression can affect the expression of miRNA‐433. For instance, in cholangiocarcinoma (CCA) cell lines, miRNA‐433 upregulation caused a reduction in *HDAC6* levels, inhibited the proliferation, colony formation ability, cellular migration, and stimulated ciliogenesis.^24^

In 2019, Weiwen Hong et al. found that in the colorectal cancer cells, the overexpression of MiRNA‐433 inhibited the epithelial‐mesenchymal transformation (EMT) process, thereby attenuating the proliferation, migration and invasion of these cells.^34^ It appears that the mechanism of action of miRNA‐433 in cancer might also have an important effect on colony forming ability, EMT, and ciliogenesis, which also has certain implications for further research related to the diverse functions of miRNA‐433.

## 4 ROLE IN REGULATING TUMOR‐RELATED FEATURES: LESION FORMATION, PATHOLOGICAL GRADING, AND TUMOR RECURRENCE

Based on the findings of various important studies, the possible relationship between gastric cancer and miRNA‐433, and the mechanism of action of miRNA‐433 in gastric cancer has become clearer，For example, in 2013, Ouy Ang Yang et al. reported that the down‐regulation of miRNA‐433 was directly related to the location and pathological grade of the lesions in gastric cancer. Overall, miRNA‐433 plays an important regulatory role in the development of gastric cancer, and changes in its expression can provide a novel basis for the diagnosis of the disease.^27^

Thereafter, in 2016, Jian Zhang et al. evaluated the impact of miRNA‐433 expression on the proliferation, adhesion, migration and invasion of colorectal cancer cells through in vitro experiments. The results showed that low expression of miRNA‐433 was associated with advanced tumor stage and early recurrence. Restoration of miRNA‐433 expression or liposome‐mediated mimetic of miRNA‐433 significantly inhibited the growth of colorectal cancer xenografts.^36^

It appears that miRNA‐433 may act as a possible tumor suppressor in colorectal cancer, and the detection of low expression of miRNA‐433 might be used as a possible biomarker to detect early recurrence of colorectal cancer patients in future studies.

## 5 MODE OF ACTION OF MIRNA‐433 INVOLVED IN ATTENUATION OF TUMORIGENESIS

**FIGURE 4:**
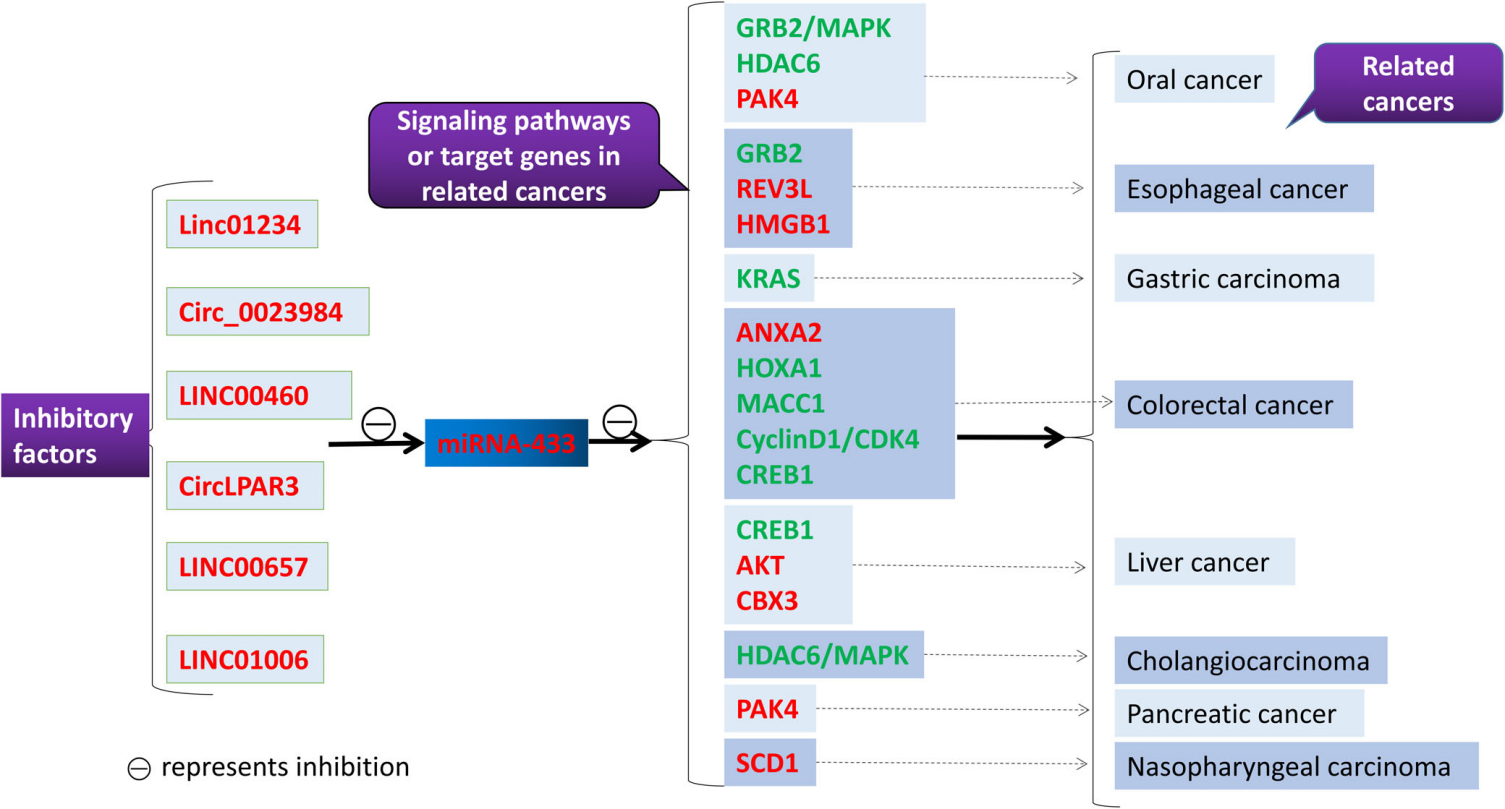
Tumorigenesis Mechanisms regulated by miRNA‐433. The upstream inhibitory factors can attenuate the inhibitory effect of miRNA‐433 on the downstream signaling pathways or the target genes by suppressing the expression of miRNA‐433, thus promoting tumor development(Both upstream inhibitory and downstream genes regulated by miR‐433 are highlighted in red.). In addition, miRNA‐433 can also attenuate the tumor development by directly inhibiting the downstream signaling pathways or the expression of the target genes(The various target genes or signaling pathways directly acted upon by miRNA‐433 are highlighted in green)

By summarizing the various articles published on the different digestive tract malignant tumors and miRNA‐433, it has been found that miRNA‐433 primarily plays a tumor suppressive role in these digestive tract malignant tumors by two distinct mechanisms (Figure 4).

First, miRNA‐433 exerts its inhibitory effect on the cancer cells by directly acting on the various downstream‐related target genes or signaling pathways in the digestive tract malignant tumors, thereby reducing their proliferation, migration and invasion capabilities. This can be also observed in some malignant tumors of the digestive tract, For example, in 2014, Xiaochun Wang et al. reported that miR‐433 exhibited a tumor suppressor role in oral cancer cells by directly targeting and downregulating the expression of *HDAC6*.^30^ In addition, in 2015, Jiaxin Li et al. used miR‐433 mimics to artificially up‐regulate miR‐433 in the colorectal cancer cell lines. The results indicated that up‐regulation of miR‐433 could significantly reduce the survival rate of colorectal cancer cells and promote apoptosis by down‐regulating *MACC1*,^35^ thus suggesting that miR‐433 might play an important role in the pathogenesis of colorectal cancer.

Second, upstream inhibitory factors, miRNA‐433, and downstream factors can potentially form a tight regulatory axis. Upstream inhibitory factors can inhibit the expression of miRNA‐433, thereby resulting in increased expression of its downstream factors, which can effectively promote the proliferation and survival of the cancer cells. For example, in 2020, Deyu Liu et al. found that Linc01234 acting as an upstream inhibitor can attenuate the expression of miRNA‐433, thereby resulting in an increase in the expression of *PAK4*, which plays a pivotal role in promoting the progression of oral cancer,^31^ which suggested that miRNA‐433 can serve as a potential therapeutic target for oral cancer. In 2021,Yaobo Song et al. found that targeted inhibition of *LINC01006* led to elevated miRNA‐433 expression, which suppressed *CBX3* expression and attenuated the proliferation, migration and invasion of hepatocellular carcinoma cells.^23^ In the same year, YuShui Ma et al. reported that in hepatocellular carcinoma cells, *KDM5A* inhibited the expression of miRNA‐433, and when *KDM5A* was silenced, the proliferation, migration, as well as invasion ability of these cancer cells were significantly reduced, and the angiogenic ability of human umbilical vein endothelial cells was also inhibited.^26^

## 6 ROLE OF MIRNA‐433 IN OTHER CANCER

As described above, we have primarily focused on the tumor suppressive role of miRNA‐433 in the digestive tract malignant tumors, but the tumor suppressive effects of miRNA‐433 are reflected in other malignancies as well. However, this paper primarily describes the reports of miRNA‐433 and some digestive tract malignant tumors to provide useful directions for the future research. Here, we also briefly tease out the role of miRNA‐433 in other important cancers.

### 6.1 Lung cancer

In 2019, Jianing Li et al. found that miRNA‐433 can inhibit the tumor progression of non‐small cell lung cancer through *SMAD family member 2(SMAD2)*.^40^ Therefore, miRNA‐433 may be a candidate molecule worthy of further exploration in the prognosis and treatment of the non‐small cell lung cancer. The article published by Lei Weng et al. indicated that miRNA‐433 could be suppressed by *PCGEM1*. In addition, WT1 associated protein *(*WTAP*)* was recognized as the downstream target of miRNA‐433, which provided a new direction for the treatment of non‐small cell lung cancer.^41^ In 2021, Furui Zhang et al. reported that miRNA‐433 overexpression significantly attenuated the proliferation and autophagy in non‐small cell lung cancer cells, but increased apoptosis.^42^ Wenshu Chen et al. first confirmed that *MED13L_012*, miRNA‐433, and *mitogen‐activated protein kinase 8(MAPK8)* can play an important role in the pathogenesis of non‐small cell lung cancer.^43^ This study is expected to provide an important step in the treatment of non‐small cell lung cancer. Boyang Liu et al. proposed that the expression of miRNA‐433 was significantly negatively correlated with the tumor size, distant metastasis, TNM stage, and poor prognosis, and proposed that miRNA‐433 can inhibit cisplatin chemoresistance by regulating DNA damage.^44^ Therefore, it appears that targeting miRNA‐433 may serve as an effective anticancer strategy.

### 6.2 Breast cancer

In 2018, Xiaolei Hu et al. first proposed that miRNA‐433 can exert a tumor suppressor effect by targeting *Serine/threonine kinase 3(AKT3)* in breast cancer.^45^ So far, the molecular mechanism of breast cancer pathogenesis is still unclear. This article undoubtedly provides a new idea for the study of the molecular mechanisms, which can regulate progression of breast cancer. Tao Zhang et al. published an article highlighting the role of miR‐433 as a tumor suppressor gene in breast cancer.^46^

In 2020, Jinhui Xue et al. studied the expression of miRNA‐433 in breast cancer serum and concluded that the survival rate of the high expression group was significantly higher than that of the low expression group.^47^ This finding suggested that miRNA‐433 could reflect the severity of breast cancer. Shiqin Liu et al first proposed that in ER+ breast cancer, miRNA‐433 can exert a tumor suppressive effects by inhibiting M2 macrophage polarization.^48^ The findings of these various studies have indicated that miRNA‐433 is worthy of attention, has tremendous research value and might provide a new direction for breast cancer treatment targeting miRNA‐433.

### 6.3 Gynecological tumors

In 2016, T. LIANG et al. published an article describing the potential relationship between ovarian cancer and miRNA‐433. The article pointed out that miRNA‐433 can inhibit the development of ovarian cancer by downregulating the expression of *notch 1(Notch1)*.^49^ The publication provided novel insights for studying the role of miRNA‐433 in the gynecological tumors. Next, Changyan Liang et al. reported that miRNA‐433 inactivated the *AKT* and ‐catenin pathways in cervical cancer, thereby acting as a tumor suppressor.^50^ This study was the first to demonstrate the tumor suppressive effect of miRNA‐433 in cervical cancer. This result was confirmed again in 2019 by Lin Ding et al., who showed that *CIRC‐ATP8A2* could effectively promote the growth of cervical cancer cells by inhibiting the expression of miRNA‐433.^51^ Based on this study, miRNA‐433 might also act as a potential therapeutic target in gynecological tumors.

### 6.4 Urinary system tumors

In 2016, the inhibition of bladder cancer EMT by miRNA‐433 was confirmed by Xu et al. Their studies indicated that miRNA‐433 exerted its tumor suppressive effects by regulating the various signaling pathways in bladder cancer.^52^ Thus, targeting the signaling pathways that by miRNA‐433 may be critical for the treatment of bladder cancer. In addition, the previous studies in 2020 and 2021 have reported that miRNA‐433 is also involved in the regulation of proliferation, migration, invasion and apoptosis of bladder cancer, and is primarily expressed as a tumor suppressor gene in bladder cancer.^53^, ^54^ These findings provides a theoretical basis for miRNA‐433 to serve as a new tumor marker for bladder cancer.

Xianguo Cai et al. studied the role of miRNA‐433 in renal cancer in 2020, and demonstrated for the first time that miRNA‐433 inhibitors can significantly reverse cell growth and migration caused by *PCGEM1* downregulation,^55^ This study explored the molecular mechanisms underlying renal carcinogenesis and revealed molecular‐mediated therapies for renal cancer.

### 6.5 Nervous system: glioma

Kai Yin et al. took the lead by analyzing the role of miRNA‐433 in glioma and concluded that miRNA‐433 could be inhibited by *CircMMP1*, thereby attenuating the inhibitory effect of miNRA‐433 on the downstream target gene *High mobility group box 3(HMGB3)* and finally promoting glioma progression.^56^ In 2021, Aiwu You et al. and Jing Zhang et al. repeated the study related to the role of miRNA‐433 in glioma, and they concluded that miRNA‐433 was inhibited upon the development of glioma.^57^, ^58^

### 6.6 Others: retinoblastoma and osteosarcoma

The similar tumor suppressive effects of MiRNA‐433 have been demonstrated in retinoblastoma^59^ and osteosarcoma,^60^, ^61^ and it was reported that miRNA‐433 acted as a potent tumor suppressor gene. These findings are consistent with the role of miRNA‐433 in other cancers. Overall, all these studies indicated that miRNA‐433 is effective, valuable and worthy of further study. It is very likely to become a new tumor marker or therapeutic target.

## 7 CONCLUDING REMARKS

In recent years, with the in‐depth research on the various miRNAs, the potential role of miRNA‐433 in different malignant tumors has been gradually revealed. It has been reported that miRNA‐433 can also play an important role in development of some non‐malignant diseases. These include myocardial ischemia‐reperfusion injury,^62^ Alzheimer's Disease (AD).^63^ polycystic ovary syndrome (PCOS),^64^ ulcerative colitis,^65^ major depressive disorder,^66^ cardiac fibrosis^67^ and acute vertigo.^68^

MiRNA‐433 can also act as a potential gallbladder carcinoma‐related miRNA.^69^ Moreover, *MAPK*, a classical signal transduction pathway was found to be up‐regulated in gallbladder carcinoma (GBC).^70^ However, the functions and mechanisms of miRNA‐433 and *MAPK* signaling pathway in GBC remain elusive.

Gallbladder carcinoma is a highly invasive biliary carcinoma with short median survival. A complete surgical resection is the only cure in patients with gallbladder carcinoma. Nevertheless, only limited patients are diagnosed early and can receive the surgical treatment and postoperative adjunctive chemotherapy.^71^, ^72^ Therefore, novel detection tools and treatment methods are particularly important for the management of this lethal disease.

After years of research on gallbladder carcinoma, our group found that the invasion and migration of gallbladder cancer cells significantly increased after overexpressing heparanase. The high expression of heparanase in gallbladder cancer cells could significantly down‐regulate the expression of syndecan‐1. In addition, *syndecan‐1* gene knockdown can promote the proliferation, invasion and metastasis of GBC cells by regulating *ERK/Snail* signal transduction and inducing EMT and cancer cells invasion.^73^, ^74^ However, whether miR‐433 plays a similar role in GBC as in other malignant tumors of digestive tract needs to be identified in the future experiments.

## AUTHOR CONTRIBUTIONS

**Jie Tang:** Data curation (lead); methodology (lead); supervision (lead); writing – original draft (lead); writing – review and editing (lead). **Jiawei Chen:** Data curation (equal); investigation (equal); writing – original draft (equal); writing – review and editing (equal). **Yongqiang Wang:** Data curation (supporting); investigation (supporting); writing – original draft (supporting); writing – review and editing (supporting). **Shaobo Zhou:** Data curation (supporting); funding acquisition (lead); methodology (supporting); writing – original draft (supporting); writing – review and editing (supporting).

## FUNDING INFORMATION

This study was funded by The Second Affiliated Hospital of Bengbu Medical College. Anhui University Natural Science Research Project (KJ2020A0564). Scientific research project of Anhui Provincial Health Commission (AHWJ2021a012). Natural Science Key Project of Bengbu Medical College (2021byzd197).

## CONFLICT OF INTEREST

The authors declare there is no conflict of interest.

## Data Availability

Data sharing not applicable as no datasets generated and/or analyzed for this study. All data relevant to the study are included in the article.
